# Towards a Safe and Effective Lithium Therapeutic Range for Older Adults With Bipolar Disorder: An ISBD Task Force Systematic Review

**DOI:** 10.1111/bdi.70151

**Published:** 2026-07-16

**Authors:** Paula V. Nunes, Andrew T. Olagunju, Debbie Zittema, Astha Dhyani, Lei Fu, Peijun Chen, Laura Montejo, Gabriel R. Fries, Lars V. Kessing, Eduard Vieta, Orestes V. Forlenza, Annemieke Dols, Hilary P. Blumberg, Lisa T. Eyler, Kenneth I. Shulman, the International Society for Bipolar Disorders (ISBD) Older Adults with Bipolar Disorder (OABD) Lithium Workgroup Task Force

**Affiliations:** ^1^ Department and Institute of Psychiatry HCFMUSP Hospital das Clínicas da Faculdade de Medicina da Universidade de São Paulo São Paulo Brazil; ^2^ Department of Medicine University of British Columbia Vancouver British Columbia Canada; ^3^ Department of Psychiatry and Behavioral Neurosciences McMaster University/St. Joseph's Healthcare Hamilton Hamilton Ontario Canada; ^4^ Department of Psychiatry Amsterdam UMC, Vrije Universiteit Amsterdam Amsterdam the Netherlands; ^5^ Amsterdam Public Health, Mental Health Research Program Amsterdam the Netherlands; ^6^ GGZ inGeest Specialized Mental Health Care Amsterdam the Netherlands; ^7^ Faculty of Science University of British Columbia Vancouver British Columbia Canada; ^8^ Precision Diagnostics and Therapeutics Program, Sunnybrook Health Sciences Centre; Department of Laboratory Medicine and Pathobiology University of Toronto Toronto Ontario Canada; ^9^ Department of Psychiatry, Geriatric Division, Case Western Reserve University School of Medicine University Hospitals Cleveland Medical Center Cleveland Ohio USA; ^10^ Geriatric Research, Education and Clinical Center (GRECC) and Psychiatric Service VA Northeast Ohio Healthcare System Cleveland Ohio USA; ^11^ Bipolar and Depressive Disorders Unit, Institute of Neuroscience University of Barcelona, Hospital Clinic, IDIBAPS, CIBERSAM Barcelona Spain; ^12^ Department of Psychiatry & Behavioral Sciences McGovern Medical School, University of Texas Health Science Center at Houston Houston Texas USA; ^13^ Faculty of Health and Medical Sciences, Department of Clinical Medicine University of Copenhagen Copenhagen Denmark; ^14^ Institute of Neuroscience University of Barcelona, Hospital Clinic, IDIBAPS, CIBERSAM Barcelona Spain; ^15^ Laboratory of Neuroscience (LIM‐27), Department and Institute of Psychiatry HCFMUSP Hospital das Clínicas da Faculdade de Medicina da Universidade de São Paulo São Paulo Brazil; ^16^ Department of Psychiatry, Division Brain University Medical Center Utrecht Utrecht the Netherlands; ^17^ Department of Psychiatry Yale School of Medicine New Haven Connecticut USA; ^18^ Department of Psychiatry University of California San Diego La Jolla California USA; ^19^ Department of Psychiatry, Sunnybrook Health Sciences Centre, Faculty of Medicine University of Toronto Toronto Ontario Canada

**Keywords:** aged, bipolar disorders, depression, drug interactions, drug monitoring, drug‐related side effects and adverse reactions, geriatric psychiatry, lithium, older adults, systematic review

## Abstract

**Objectives:**

Lithium is recommended as the first‐line maintenance treatment for older adults with bipolar disorder (OABD). However, age‐related reduction in renal clearance, heightened sensitivity to adverse effects, and drug interactions increase the risk of toxicity and necessitate lower therapeutic serum levels in this population. Despite expert consensus supporting age‐adjusted targets, most clinical laboratories still do not report age‐specific therapeutic ranges. We conducted a systematic review on recommended serum lithium levels in OABD to update evidence supporting age‐specific therapeutic ranges for safe and effective lithium management.

**Methods:**

We searched Medline, PsycINFO, Embase, and Cochrane Central Register of Controlled Trials since 2017 for studies analyzing lithium serum levels in individuals with bipolar disorder ≥ 60 years. Two reviewers independently screened titles, abstracts, and full texts. We used the National Institutes of Health Quality Assessment Tool to assess methodological quality.

**Results:**

Fourteen studies, including 8808 older adults using lithium, met inclusion criteria. All studies were observational with cross‐sectional or cohort designs. Mean or median lithium levels ranged from 0.47–0.67 mmol/L. Levels of 0.82–1.00 mmol/L were associated with increased side effects or early toxicity signs, while levels of 1.20–1.25 mmol/L were overtly toxic. Impaired renal function and concomitant use of diuretics, ACE inhibitors, and NSAIDs required closer monitoring for toxicity.

**Conclusions:**

These findings are consistent with age‐adjusted lithium therapeutic targets in older adults and provide additional observational support for clinical laboratories to adopt age‐specific therapeutic ranges. Clinicians should consider individualized monitoring accounting for age‐related physiological changes and medication interactions to ensure safer and effective lithium therapy.

## 1 Introduction

Lithium remains the first‐line medication for the maintenance treatment of bipolar disorder (BD) [1, 2], with established recommendations extending to older adults living with BD (OABD) [1, 3, 4, 5]. The community prevalence of BD is approximately 1%–2% in both younger and older populations [6, 7], but among clinical inpatients aged 50 years or older, the prevalence can be considerably higher, up to 6% [8]. Multi‐site epidemiological studies indicate that nearly half of OABD use lithium for maintenance treatment [9, 10]. Despite its well‐established benefits including reducing depressive and manic episodes, preventing relapse [2, 11], and offering potential neuroprotective effects [5] and reduced suicide risk [12, 13, 14], lithium requires close monitoring due to its narrow therapeutic index [12, 15, 16] and potential side effects [17]. As a result, the serum levels needed for clinical benefit [18] can be close to toxic levels, with resulting short‐term and long‐term life‐threatening effects. In order to mitigate this risk, close monitoring of lithium serum levels is easily available in most services, and can support clinical decision‐making [11, 15, 16]. When properly managed, lithium is not associated with an increased somatic burden such as non‐psychiatric admissions [2], cardiovascular disease, diabetes mellitus or even chronic kidney disease compared to alternative treatments [2, 19]. Despite potential cognitive side effects for some individuals, lithium is not associated with an increased risk of dementia or other neurocognitive disorders, and may have neuroprotective properties [20, 21]. Moreover, OABD using lithium have been shown to express a more positive attitude towards psychotropic pharmacotherapy, despite high rates of adverse effects [22]. Therefore, the decrease in lithium prescriptions observed over the last few decades in many countries [23, 24, 25], but not all [26, 27, 28], is not based on the evidence and best practice.

Older adults require closer monitoring due to an age‐related decrease in renal clearance (pharmacokinetics) and heightened sensitivity (pharmacodynamics) to lithium side effects. These include tremor and other Parkinsonian symptoms as well as an increased risk of cardiovascular comorbidities [4, 6, 29]. Additionally, OABD are vulnerable to drug interactions with commonly used medications such as diuretics, angiotensin‐converting enzyme (ACE) inhibitors, and non‐steroidal anti‐inflammatory drugs (NSAID) that can raise lithium levels precipitously [4, 6, 29, 30]. Consequently, previous publications support lower lithium treatment doses for this population [29, 31, 32, 33, 34]. For adults, meta‐analysis supports serum lithium ranges between 0.6 and 1.2 mmol/L for the prophylaxis of any type of episode, and usually lithium levels for acute mania tend to be higher [35]. In the absence of systematic data on older adults and the challenge of conducting randomized clinical trials in this population [36], an expert panel of the International Society for Bipolar Disorders (ISBD) conducted a Delphi survey and recommended for maintenance treatment serum levels of 0.4–0.8 mmol/L for individuals aged 60–79 years and serum levels of 0.4–0.7 mmol/L for those aged 80 years and over [3]. Similarly, another consensus panel composed of German specialists proposed an even lower therapeutic range of 0.4–0.7 mmol/L for individuals aged 60–79 years and serum levels of 0.4–0.6 mmol/L for those aged 80 years and over [37]. While these recommendations differ slightly in upper thresholds, both support substantially lower levels than those recommended for younger adults.

Most geriatric psychiatrists are aware of the benefit of using lower serum levels for maintenance treatment of OABD. However, general psychiatrists and general practitioners, who out of necessity are responsible for the clinical care of OABD, are often not aware of the recommended lower therapeutic levels [38], and still rely on existing (and not yet updated) laboratory‐reported levels to guide their practice. Unfortunately, the vast majority of laboratories, with a few notable exceptions, still do not report the therapeutic range for serum levels of lithium separately for older adults [39]. Given the gap between expert consensus recommendations and clinical laboratory reporting practices, this systematic review aims to update and synthesize the evidence on serum lithium levels in OABD from 2017 onward, complementing earlier work published in 2018 [40] and 2019 [41] on lithium maintenance therapy. Specifically, we examined therapeutic serum levels, toxicity thresholds, and factors influencing lithium safety in this population. Our findings seek to provide an evidence base that reinforces expert recommendations for age‐specific therapeutic ranges and supports the implementation of age‐adjusted reference values in clinical laboratories, ultimately promoting safer and more effective lithium therapy for older adults.

## 2 Methods

We reported a narrative synthesis of this systematic review in accordance with the Preferred Reporting Items for Systematic Reviews and Meta‐analyses (PRISMA) 2020 reporting guideline [42] and registered the protocol with PROSPERO (CRD420251054503) before starting the review.

### 2.1 Search Strategy

A librarian at the University of British Columbia (Edlyn Lim) guided an electronic search of peer‐reviewed literature in English on May 29, 2025, for studies published since 2017 to update earlier work on lithium maintenance therapy [40, 41]. The following databases were chosen: Medline (Ovid), Embase (Ovid), PsycINFO (EBSCO), and Cochrane Central Register of Controlled Trials for studies.

The search strategy incorporated MeSH terms and/or keywords including lithium, BD and older adults, geriatric, aged, senior or elders. Data S1 provides further details on the search methods. After removing duplicates, two independent reviewers (Paula V. Nunes and Astha Dhyani) screened titles and abstracts; disagreements were resolved through discussion. Any residual discrepancies were resolved by consultation with a third reviewer (Kenneth I. Shulman). The same reviewers then conducted full‐text reviews to assess eligibility following the same conflict resolution process.

### 2.2 Eligibility Criteria and Study Selection

Studies were included if they: (a) exclusively enrolled adults ≥ 60 years to facilitate comparison with previous consensus guidelines [3, 37], (b) provided separate age‐stratified analyses for adults ≥ 60 years, or (c) included age as a variable with sufficient data to extract outcomes specific to older adults. The primary outcome of interest was serum lithium levels for maintenance treatment. Secondary outcomes of interest included lithium daily doses, tolerability, toxicity, and interaction with other medications. Case studies, case series, reviews, book chapters, or poster abstracts were excluded. Lithium prescribing trends, studies where the therapeutic effect of lithium was not tested, or studies that did not have data on lithium serum levels were also excluded. All review processes were conducted using the web‐based software Covidence.

### 2.3 Methodological Quality Assessment

The methodological quality of included cohort and cross‐sectional observational studies was evaluated (Andrew T. Olagunju) using the National Institutes of Health (NIH) Quality Assessment Tool based on multiple criteria [43, 44].

### 2.4 Data Extraction and Synthesis

Data extracted from the final set of articles included author names, publication year, country, study description (e.g., study design, study setting/location, sample size, and demographics), patient age and demographics, psychiatric diagnosis and comorbidities, lithium serum levels and range, daily lithium doses, and overall findings relevant to this review (e.g., test statistic, confidence interval, prevalence). We also qualitatively assessed clinical outcomes (e.g., efficacy, tolerability, toxicity, mortality), and factors examined as potential mediators, moderators, or confounders (e.g., renal function, comorbid medical conditions, polypharmacy, cognitive status). Data were organized into thematic categories by lithium level range, clinical outcome, and population subgroup. Statements regarding variations across studies (e.g., differences in target serum level thresholds, lithium monitoring frequency, outcome measurement approaches) reflected qualitative assessment of the range of reported characteristics, not statistical testing. Dose–response relationships were identified when individual studies reported and tested graded associations between lithium serum levels and clinical or safety outcomes. Comparisons by age subgroup (e.g., young‐old vs. old‐old) or care setting were based on individual studies that examined these distinctions, not on cross‐study effect size comparisons. Data extraction was performed by one reviewer (Paula V. Nunes) with verification by a second (Astha Dhyani).

## 3 Results

**FIGURE 1:**
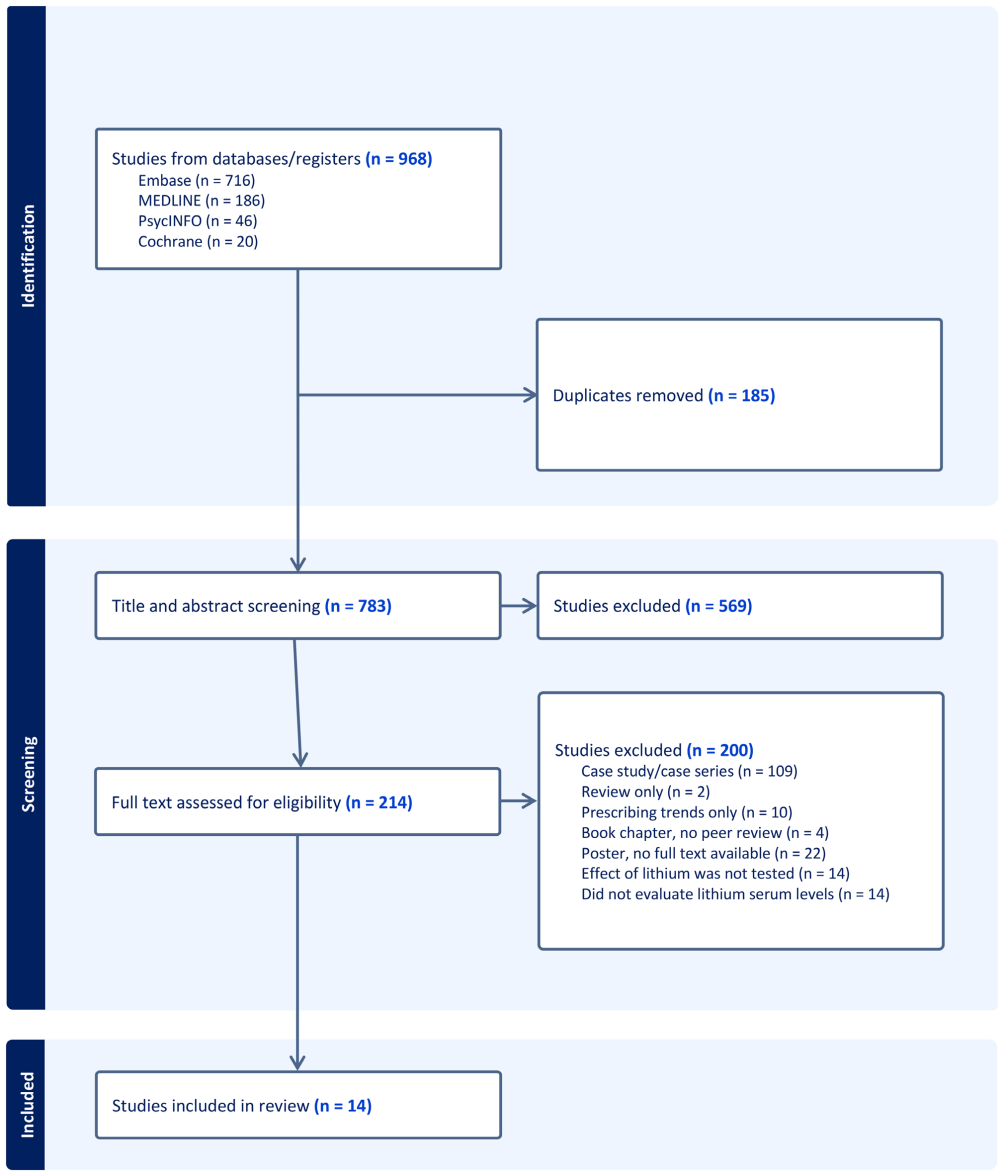
PRISMA flow chart summarizing the screening process.

The search strategy yielded 968 articles. After removing duplicates, 783 titles and abstracts were screened, of which 569 were excluded. A total of 214 full‐text articles were assessed for eligibility, with 200 excluded for reasons detailed in the PRISMA flow chart (Figure 1). Finally, 14 studies met inclusion criteria and, after quality of bias assessment (Table 1), were included in this systematic review [45, 46, 47, 48, 49, 50, 51, 52, 53, 54, 55, 56, 57, 58].

**TABLE 1:** National Institutes of Health Quality Assessment Tool quality assessment of observational cohort and cross‐sectional studies included studies (*n* = 14).

Study	1	2	3	4	5	6	7	8	9	10	11	12	13	14	Overall rating
Bocchetta et al., 2017 [45]	Yes	Yes	Yes	Yes	No	Yes	Yes	No	Yes	Yes	Yes	N/A	No	Yes	Good
Brouwer et al., 2022 [46]	Yes	Yes	Yes	Yes	No	N/A	Yes	N/A	Yes	N/A	Yes	N/A	N/A	Yes	Fair
Burton et al., 2021 [47]	Yes	Yes	CD	Yes	No	N/A	Yes	No	Yes	No	Yes	N/A	CD	No	Fair
Carli et al., 2022 [48]	Yes	Yes	Yes	Yes	No	No	N/A	No	Yes	No	Yes	N/A	CD	No	Fair
Chen et al., 2022 [49]	Yes	Yes	Yes	Yes	No	Yes	Yes	Yes	Yes	Yes	Yes	Yes	CD—	Yes	Good
Dinnen et al., 2017 [50]	Yes	Yes	Yes	Yes	No	CD	Yes	No	Yes	Yes	Yes	N/A	CD	No	Fair
Flapper et al., 2021 [51]	Yes	Yes	Yes	Yes	No	N/A	Yes	No	Yes	N/A	Yes	Yes	Yes	Yes	Good
Fransson et al., 2025 [52]	Yes	Yes	Yes	Yes	No	Yes	Yes	No	Yes	No	No	N/A	Yes	Yes	Good
Ganter et al., 2019 [53]	Yes	Yes	Yes	Yes	No	Yes	Yes	Yes	No	Yes	Yes	N/A	CD	No	Good
Golic et al., 2021 [54]	Yes	Yes	Yes	Yes	No	Yes	Yes	Yes	Yes	Yes	Yes	N/A	CD	Yes	Good
Kraszewska et al., 2019 [55]	Yes	Yes	CD	Yes	No	No	Yes	Yes	Yes	No	Yes	N/A	CD	No	Fair
Nederlof et al., 2019 [56]	Yes	Yes	Yes	Yes	No	N/A	Yes	No	Yes	Yes	Yes	N/A	CD	N/A	Good
Rej et al., 2018 [57]	Yes	Yes	Yes	Yes	CD	CD	Yes	Yes	Yes	Yes	Yes	N/A	CD	Yes	Good
Scherf‐Clavel et al., 2020 [58]	Yes	Yes	CD	Yes	No	N/A	Yes	No	N/A	No	Yes	N/A	CD	Yes	Fair

*Note:* Quality assessment items' criteria: (1) Were the research questions or objective in this paper clearly stated? (2) Was the study population clearly specified and defined? (3) Was the participation rate of eligible persons at least 50%? (4) Were all the subjects selected or recruited from the same or similar populations (including the same time period)? Were inclusion and exclusion criteria for being in the study prescribed and applied uniformly to all participants? (5) Was a sample size justification, power description, or variance and effect estimates provided? (6) For the analyses in this paper, were the exposure(s) of interest measured prior to the outcomes(s) being measured? (7) Was the timeframe sufficient so that one could reasonably expect to see an association between exposure and outcome if it existed? (8) For exposures that can vary in amount or level, did the study examine different levels of the exposure as related to the outcome (e.g., categories of exposure, or exposure measured as continuous variable)? (9) Were the exposure measures (independently variables) clearly defined, valid, reliable, and implemented consistently across all study participants? (10) Was the exposure(s) assessed more than once over time? (11) Were the outcome measures (dependent variables) clearly defined, valid, reliable, and implemented consistently across all study participants? (12) Were the outcome assessors blinded to the exposure status of participants? (13) Was loss to follow up after baseline 20% or less? (14) Were key potential confounding variables measured and adjusted statistically for their impact on the relationship between exposure(s) and outcomes(s)?

Abbreviations: CD, cannot determine; N/A, not applicable.

### 3.1 Quality Assessment

Eight studies (57.1%) received an overall rating of “good,” and six (42.9%) received an overall rating of “fair.” No studies were rated as “poor.”

### 3.2 Study Characteristics

Detailed information on the included studies is displayed in Table 2. Studies were observational, employing retrospective cohort [46, 47, 48, 49, 50, 51, 52, 54, 56], longitudinal cohort [45, 57] or cross‐sectional designs [53, 55, 58]. Studies addressed diverse research objectives. One study compared lithium with second‐generation antipsychotics for older adults [47], while five examined lithium monitoring compliance [46, 56, 57] and therapeutic ranges for treatment [48] and toxicity [53]. One study investigated reasons for lithium discontinuation [51], four investigated renal function and chronic kidney disease [45, 50, 52, 54], endocrine and two studies metabolic complications [50, 55], one study investigated drug interactions [58], and one study analyzed the neuroprotective effects of lithium [49].

**TABLE 2:** Summary of included studies (*n* = 14).

Study	Country	Study design—observational	Diagnosis	Lithium sample size for older adults	Age (years), mean or median (±SD or IQR)	Age range	Female	Adopted therapeutic levels for older adults (mmol/L)	Lithium therapeutic level (mmol/L) mean or median (±SD or IQR)
Bocchetta et al., 2017 [45]	Italy	Cohort	BD (81%), MDD (18%)	110	72 (NG)	65–84	73%	0.40–0.80	0.55 (NG)
Brouwer et al., 2022 [46]	Netherlands	Cohort, retrospective	BD (47%), MDD (26%), SCZA (11%), unspecified (16%)	129	60 (NG)	23–87	57%	0.40–1.20	NG
Burton et al., 2021 [47]	United States	Cohort, retrospective	BD	24	71 (NG)	65–87	4%	NG	NG
Carli et al., 2022 [48]	Italy	Cohort, retrospective	“Mostly BD”	NG	NG	65+	NG	0.50–0.80	0.47 (±0.31)
Chen et al., 2022 [49]	United Kingdon	Cohort, retrospective	NG	548	73 (±12)	50+	59%	NG	0.61 (0.49–0.80)
Dinnen et al., 2017 [50]	Ireland	Cohort, retrospective	NG	580	55 (44–65)	NG	42%	NG	NG
Flapper et al., 2021 [51]	Netherlands	Cohort, retrospective	BD (26%), MDD (57%), SCZA or SCZ (17%)	135	69 (63–76)	60+	92%	NG	0.60 (NG)
Fransson et al., 2025 [52]	Sweden	Cohort, retrospective	BD (81%), MDD (11%), SCZA (8%), unspecified (16%)	168	62 (±14)	24–91	56%	NG	0.61 (±0.13)
Ganter et al., 2019 [53]	Ireland	Cross‐sectional	BD (39%), MDD (14%), SCZA (7%), unspecified (41%)	12	NG	65+	48%	0.60–0.80	NG
Golic et al., 2021 [54]	Sweden	Cohort, retrospective	NG	166	61 (±15)	NG	61%	NG	0.58 (±0.11)
Kraszewska et al., 2019 [55]	Poland	Cross‐sectional	BD	98	62 (±13)	24–85	69%	0.50–0.80	NG
Nederlof et al., 2019 [56]	Netherlands	Cohort, retrospective	NG	834	57 (±17)	56+	60%	NG	NG
Rej et al., 2018 [57]	Canada	Cohort	NG	5503	68 (66–74)	65+	59%	0.40–0.80	NG
Scherf‐Clavel et al., 2020 [58]	Germany	Cross‐sectional	BD (60%), MDD (30%), SCZA (10%)	501	49 (±22)	18–89	55%	NG	0.67 (±0.25)

Abbreviations: BD, bipolar disorder; IQR, interquartile range; MDD, major depressive disorder; NG, not given; SCZ, schizophrenia; SCZA, schizoaffective disorder; SD, standard deviation.

Studies included approximately 8808 older adults in total, with sample sizes ranging from 12 to 5503 (median = 142). This represents a conservative estimate as one study [48] reported measurements without specifying unique patient numbers contributing to these data. The sample‐size weighted mean age of older adult participants was 64.9 ± 7.5 years. Two studies did not report age information [48, 53]. No studies provided separate analyses for the oldest‐old (typically defined as ≥ 70 years) [10], and only one study [57] included this subgroup in baseline characteristic tables. Gender or sex distribution varied across studies, with women/female comprising 42%–73% of participants in most studies. Two notable exceptions included a study of United States Veterans, with 4% of women, and another study with 92% of women [51]; for the latter, 57% of participants had major depressive disorder (MDD). Only two studies were conducted outside Europe: one in the United States [47], and one in Canada [57]. No studies from other continents met inclusion criteria. Three studies included exclusively or predominantly individuals with BD [47, 48, 55]. In the remaining studies with diagnostic information available, BD was the most common diagnosis, followed by MDD and schizoaffective disorder [45, 46, 52, 53, 58], except for one, where MDD was more prevalent than BD [51]. Most studies focused exclusively on older adults [45, 47, 48, 49, 51, 53, 56, 57], while others were population‐based studies that included age‐stratified analyses or statistical adjustment for age [46, 50, 52, 54, 55, 58].

### 3.3 Lithium Levels and Dosing

A synthesis of serum lithium levels in older adults can be seen in Table 3. The reported mean or median lithium levels from the studied samples ranged from 0.47 to 0.67 mmol/L [45, 48, 49, 51, 52, 54, 58]. Levels associated with increased toxicity ranged from 0.82 to 1.00 mmol/L [50, 53], and toxic levels ranged above 1.20–1.25 mmol/L [46, 53]. Symptoms of toxicity included nausea, vomiting, diarrhea, coma, need for dialysis, hospital or intensive care unit admission. Additionally, some studies reported levels considered to be within the therapeutic range: for studies whose age range started above 60 years, therapeutic levels were considered to be 0.40–0.80 mmol/L [45, 57], 0.50–0.80 mmol/L [48, 55], or 0.60–0.80 mmol/L [53]. Two studies reported the average therapeutic daily lithium carbonate dose for 60 years or more as 450mg [45] and 560 mg [47].

**TABLE 3:** Serum lithium levels in older adults: observed ranges, clinical targets, and toxicity thresholds across included studies.

Category	Serum lithium levels (mmol/L)	Basis for category
Observed mean/median levels in study samples	0.47–0.67	Reported central tendency of measured serum levels across included study samples; reflects levels patients were maintained on in practice [10, 45, 48, 51, 52, 54, 58]
Therapeutic target ranges applied at study sites	0.40–0.80	Ranges explicitly adopted by clinicians at specific study sites as treatment targets for older adults; varied across sites (0.40–0.80, 0.50–0.80, and 0.60–0.80 mmol/L) [45, 48, 53, 55, 57]
Levels associated with increased side effects or early signs of toxicity	0.82–1.00	Levels at which individual studies reported statistically significant associations with adverse effects or early toxicity indicators [50, 53]
Levels associated with overt toxicity	≥ 1.20–1.25	Levels at which individual studies reported frank toxicity; based on observational data [46, 53]

### 3.4 Factors Associated With Lithium Concentrations and Clinical Outcomes

Associations of lithium concentrations with other variables were also explored in some studies. NSAID use was significantly associated with higher lithium levels, whereas diuretics, ACE inhibitors, and angiotensin receptor blockers were not [58]. In this study, the more medications prescribed and the lower the estimated glomerular filtration rate (eGFR), the higher was the lithium concentration. In another study, patients using medications like ACE inhibitors, angiotensin receptor blockers, or thiazides required significantly lower mean daily doses of lithium to maintain the same levels compared to those not on these medications [45]. Similar results of associations of high lithium concentrations and lower eGFR were also found in other studies [50, 53]. Finally, higher lithium concentrations were associated with increased need for hospitalization for non‐psychiatric medical reasons [53].

## 4 Discussion

### 4.1 Serum Lithium Levels and Dosing in Older Adults

This systematic review, based on 14 observational studies encompassing 8808 individuals, highlights the importance of age‐specific lithium serum levels for maintenance treatment of OABD. The observed lithium levels for older adults ranged from 0.47 to 0.67 mmol/L, which are substantially lower than the standard therapeutic range of 0.6–1.2 mmol/L, typically reported by laboratories that do not apply age‐adjusted reference ranges [39]. The observed levels of 0.47–0.67 mmol/L align closely with expert consensus [1, 3] and recent narrative reviews' recommendations [32] of 0.4–0.8 mmol/L for individuals aged 60–79 years and 0.4–0.7 mmol/L for those aged 80 years and over or even 0.4–0.6 mmol/L for adults aged 65 or older [59]. Some studies in the current review also reported therapeutic target ranges for older adults [45, 48, 53, 55, 57], and they varied from 0.40–0.80 mmol/L to 0.60–0.80 mmol/L, reflecting emerging clinical recognition of the need for age‐adjusted dosing. Levels associated with toxicity risk (0.82–1.00 mmol/L) [50, 53] and overt toxicity (≥ 1.20 mmol/L) [46, 53] in older adults remained notably lower than toxicity thresholds in younger populations, underscoring the heightened vulnerability of this age group. High serum lithium levels (above 1.5 mmol/L) were associated with increased need for non‐psychiatric hospital admission due to adverse reactions in one included study [53], a finding that may warrant consideration of this threshold as “critical” level in laboratory reporting, subject to replication. Additionally, older adults achieved therapeutic levels with lower daily lithium doses (450–560 mg) [45, 47] than typical adult maintenance doses (900–1200 mg) [60]. These findings add to the observational evidence and empirical support for implementing age‐specific lithium maintenance treatment reference ranges in clinical laboratories, and are in keeping with existing expert consensus recommendations. They also reinforce the importance of considering individualized monitoring approaches that account for age‐related pharmacokinetic and pharmacodynamic changes, reduced renal function, and common medication interactions in older adults.

### 4.2 Lithium Levels for Acute Mania

The focus of this systematic review was on reference levels for maintenance lithium treatment. However, it should be noted that lithium levels for acute mania may need to be higher. One of the few randomized controlled trials that analyzed lithium use for older adults in acute mania showed that lithium serum levels of 0.80–0.99 mmol/L demonstrated comparable efficacy and tolerability to divalproex [61]. Notably, data remain limited on the treatment of mania in OABD. A systematic review [62] published in 2017 suggests that lithium is effective and well‐tolerated, and thus should remain a first‐line treatment for acute mania in accordance with more recent guidelines [1]. It is important to note, however, that this recommendation is largely extrapolated from general adult data, given the scarcity of trials conducted specifically in older adult populations. For older adults, lithium appears more effective at lower doses with corresponding lower serum levels. Therefore, close monitoring of serum concentrations is essential. The acute treatment of mania nowadays rarely consists of lithium monotherapy, either in middle‐aged BD or OABD, as high lithium doses can carry substantial tolerability issues. Moreover, antipsychotics may act faster and more effectively in the short‐term [63]. This is a further reason to focus on lithium levels for maintenance rather than mania.

### 4.3 Monitoring Compliance

Overall compliance with regular monitoring for lithium, creatinine, calcium, and thyroid‐stimulating hormone (TSH) was found to be suboptimal [46, 50, 53, 56, 57], despite being universally recommended (e.g., by the National Institute for Health and Clinical Excellence—NICE) for avoiding toxicity as well as renal impairment [38]. Since OABD are especially vulnerable to decreased renal clearance and drug interactions, lower, age‐specific reference ranges and closer adherence to monitoring guidelines are likely associated with safer management of OABD. In this review, studies conducted in community and ambulatory settings consistently documented low monitoring rates. In a population‐based study of 5503 older lithium users in Ontario, Canada [57], researchers found that monitoring was infrequent compared to the recommended 3‐month standard for geriatric patients. Only 24.1% of patients had a serum lithium concentration recorded within 90 days, increasing to 42.4% within 180 days and 66.8% within a year. Similarly, serum creatinine was monitored within 90 days in only 29.6% of the cohort. While TSH and calcium testing rates were statistically higher in lithium users than in a control group, only 13.3% of lithium users received the recommended annual calcium test. A study analyzing 1583 ambulatory patients in the Netherlands [56] found that lithium serum levels were monitored in only 65% of the 6‐month periods of use. Creatinine was measured in 73% of periods and TSH in 54%. Most notably, only 16% of patients were fully compliant with the guidelines for all three parameters (lithium, creatinine, and TSH) throughout their entire follow‐up period. Also in the Netherlands, among 129 patients coming from community settings [46], physicians monitored lithium levels every 6 months for 55% of patients, and kidney function for 45%. Even in more specialized tertiary settings, monitoring remained below recommended standards. Analyzing a 14‐year database of 580 patients in Ireland with at least one lithium measurement [50], this study reported that the overall median number of lithium measurements per patient per year was one. While 100% of the study population had serum creatinine measured at some point during the 14‐year follow‐up, TSH was measured in 97% and serum calcium in only 86.7% of the cohort. In a slightly different approach, in a tertiary referral center in Ireland, researchers investigated retrospectively individuals with higher serum lithium levels (> 1.0 mmol/L) [53], and among the 44 identified patients (25% of those aged ≥ 65 years), 29.5% had no renal function test performed at the time their lithium levels were in the toxic range—a particularly concerning finding given the well‐established renal vulnerability of older adults. Among patients with the highest lithium levels (≥ 1.5 mmol/L), only 62.9% had a renal function test within the preceding 6 months. Taken together, these findings indicate that inadequate monitoring and the absence of age‐adjusted reference ranges represent compounding and potentially preventable risks for older adults receiving lithium therapy.

### 4.4 Lithium Levels and the Relationship With Renal Function and Risk of Kidney Impairment

To establish a safe therapeutic range for OABD with respect to kidney health, multiple factors influencing renal function must be considered, including duration of lithium exposure, maintenance serum levels, and episodes of intoxication. Episodes of intoxication may in part be precipitated by impaired maximal urine concentrating capacity (MUCC), which predisposes to dehydration—a recognized complication in individuals receiving long‐term lithium therapy—and can thereby raise the risk of toxic serum levels.

The extent to which lithium exposure affects long‐term kidney function remains unclear, particularly in OABD. The observational studies included in this review were limited by small sample sizes, retrospective designs, and either short or highly variable follow‐up periods within cohorts [45, 50, 52, 54]. The annual decline in kidney function during lithium therapy ranged from −1.1 to −2.3 mL/min/1.73 m^2^, with the steepest decline observed in the smallest cohort (*n* = 48) [45]. For context, age‐related annual eGFR decline in the general population is estimated at −0.4 to −1.1 mL/min/1.73 m^2^ per year [64], and appears to be more strongly influenced by baseline eGFR than by age, with slower decline in individuals with pre‐existing renal impairment [65]. Golic et al. [54] found no significant difference in annual eGFR decline between patients with normal kidney function (*n* = 83; baseline eGFR 80 mL/min/1.73 m^2^; annual decline −1.5 mL/min/1.73 m^2^; mean lithium level 0.58 mmol/L) and those with impaired kidney function (*n* = 83; baseline eGFR 48 mL/min/1.73 m^2^; annual decline −1.1 mL/min/1.73 m^2^; mean lithium level 0.54 mmol/L). Nonetheless, individuals with lower baseline eGFR reached clinically significant chronic kidney disease more rapidly. In this cohort, older age and greater somatic comorbidity—particularly cardiovascular disease—were associated with faster decline in kidney function. Interpretation of these findings is complicated by the wide variability in eGFR decline (−15.4 to +32.7 mL/min/1.73 m^2^). No effect of treatment duration, lithium exposure, or intoxications was observed. However, faster kidney function decline was noted among individuals with a history of prior lithium treatment—in some cases initiated one to two decades earlier—during periods when higher serum levels were more commonly targeted. Bocchetta et al. [45] reported a cross‐sectional association between treatment duration and eGFR but did not observe this association during follow‐up, which the authors attributed to limited sample size. Fransson et al. [52] examined eGFR decline during lithium treatment and following lithium discontinuation. During treatment (*n* = 168; mean lithium level 0.61 mmol/L), the annual decline was −1.58 (−1.87 to −1.28) mL/min/1.73 m^2^. After discontinuation, the decline slowed markedly to −0.023 (−0.49 to +0.44) mL/min/1.73 m^2^ per year, a pattern that persisted for at least 5 years. Upon lithium reinstatement, the rate of decline returned to levels comparable to those observed during the initial treatment period (−1.71 [−2.08 to −0.96] mL/min/1.73 m^2^). This study did not account for cumulative lithium exposure or episodes of intoxication. A recent prospective cohort study (not included in this review due to a sample mean age of 51 years, below the ≥ 60‐year inclusion threshold) found a median annual eGFR decline of 0.8 mL/min/1.73 m^2^ in 196 patients, with faster decline associated with higher lithium levels and longer treatment duration [66]. These findings support those in this review linking lithium exposure to kidney function decline. Dineen et al. [50] examined intoxications in individuals receiving lithium treatment and reported an association between intoxication episodes and the development of chronic kidney disease (CKD) and hypernatremia, possibly related to impaired urine concentrating capacity, although this pathway was not directly assessed. Notably, intoxications were not reported in the prospective cohort study described above.

None of the studies in this review evaluated MUCC, although an impairment is seen in 40%–60% of lithium‐treated patients, sometimes leading to arginine vasopressin resistance, previously known as nephrogenic diabetes insipidus [66, 67, 68]. Assessment of urine concentrating capacity is technically challenging. Self‐reported symptoms such as polyuria and polydipsia correlate poorly with objective measures [69]. Formal assessment of MUCC requires either a water deprivation test or a desmopressin stimulation test. These tests are technically demanding and impractical in routine psychiatric outpatient care; as a result, data on MUCC are seldom captured in observational studies. Nevertheless, impaired concentrating capacity can substantially affect quality of life, with notable interference in daily activities, sleep, and work‐related functioning [70, 71]. Furthermore, individuals on lithium treatment are at risk of dehydration, which may be severe, particularly older adults, in whom thirst sensation is often diminished [71, 72]. Dehydration may precipitate lithium intoxication, which in turn can accelerate renal function decline [73, 74, 75]. This cascade is of particular clinical relevance in older adults, who are disproportionately vulnerable at each stage of this pathway. Ganter et al. [53] found a higher number of intoxication episodes among individuals aged < 65 years (*n* = 63) compared to those aged ≥ 65 years (*n* = 17) in a tertiary care setting. Possible explanations include more cautious prescribing in older adults, prior lithium discontinuation in some older adults reducing the pool at risk, or more frequent healthcare contact among older adults enabling earlier detection and management of toxicity. A further plausible explanation, and one most directly relevant to this review, is the intentional use of lower therapeutic lithium target ranges in OABD, which may reduce the likelihood of reaching toxic concentrations. A notable limitation of this study is the absence of pre‐intoxication lithium level data, which precludes direct evaluation of this explanation. These findings also suggest that the clinical consequences of intoxication may be more severe in older adults: following an intoxication episode, eGFR was lower in individuals aged ≥ 65 years (53 vs. 66 mL/min/1.73 m^2^), which may in part reflect lower baseline renal function in this group (59 vs. 75 mL/min/1.73 m^2^). These findings reinforce the importance of preventing intoxication in older adults, in whom the functional renal reserve available to absorb the impact of a toxic episode is likely more limited.

With respect to the risk of renal impairment in older adults receiving lithium treatment, the available evidence does not consistently support accelerated eGFR decline in OABD when serum levels are maintained within lower therapeutic ranges, consistent with the mean levels of approximately 0.5–0.6 mmol/L observed across the studies included in this review. A lower baseline eGFR does not appear to result in faster decline, although it does increase the likelihood of reaching thresholds of clinically significant renal impairment more rapidly. Treatment duration also appears to be a meaningful contributor to cumulative renal risk, independent of serum level alone. Impaired urine concentrating capacity may contribute to dehydration and thereby increase the risk of intoxication episodes, particularly in older adults. However, intoxication episodes have not been observed at consistently higher rates in OABD compared to younger adults, and when they do occur, the impact on renal function may be disproportionately greater in this group. Taken together, these findings are consistent with the use of lower therapeutic lithium target ranges in OABD and suggest that this may represent a clinically reasonable approach to mitigating renal risk in this population.

### 4.5 Drug Interactions

On the impact of other drugs on lithium serum levels, the most detailed quantitative analysis utilized linear regression to determine how specific drug classes and the total number of medications affect lithium concentrations [58]. The number of potentially interacting drugs was significantly associated with increasing lithium serum levels, with each additional interacting drug raising the absolute concentration by approximately 0.043 mmol/L. Notably, NSAIDs were identified as the only drug class that was associated with increased lithium levels independently of other factors, raising them by an average of 0.121 mmol/L, which represents nearly 17% of the entire therapeutic range. The clinical impact of “theoretically contraindicated” medications was also examined [41]; 52% of the older adult cohort were regularly taking ACE inhibitors (e.g., enalapril), Sartans (e.g., losartan), or thiazides (e.g., hydrochlorothiazide) [45]. Clinicians appeared to adjust for these interactions by prescribing significantly lower mean daily lithium doses (384 mg) for patients on these medications compared to those not on them (464 mg). The role of co‐prescribed medications in episodes of lithium toxicity was further investigated [53]. Specific cases were identified where the addition of a thiazide diuretic led to toxic serum levels as high as 3.2 mmol/L. Co‐prescription of interacting medications, particularly ACE inhibitors and diuretics, was identified as significantly associated with toxic serum levels.

### 4.6 Tolerability and Reasons for Discontinuation

When analyzing reasons for discontinuation of lithium treatment, intoxication was cited by some papers [47, 51, 52]. In a mirror‐image study of a cohort of 168 participants focused on kidney function [52], 7% discontinued lithium following a toxic serum event. In this study, renal causes (decreasing kidney function, polyuria) were the primary driver for stopping. In a recent study [76], older age and higher lithium levels were independent predictors of kidney failure at 10‐year follow‐up. Other reasons cited were lack of effect (14%), fear of future side effects (9%), and non‐adherence (8%). In a retrospective study of 135 participants [51], lithium discontinuation was attributed to intoxication in 2.2% of cases and to reduced eGFR in 8.2%. Clinically relevant adverse effects contributing to discontinuation included tremor (18.4%) and gait disturbance (10.2%). Finally, lack of effectiveness (34.7% of all discontinuers) and noncompliance (26.5% of all discontinuers) were also observed. Interestingly, in a study on older veterans with BD which aimed to compare time to discontinuation between lithium users (*N* = 191) and second‐generation antipsychotics (*N* = 1181) [47], no difference was found between the groups. For lithium, toxicity, changes in renal function, and tremors were among the reasons for discontinuation.

Other studies also compared non‐renal side‐effects. With regards to thyroid function, lithium users, when compared with those using other mood‐stabilizing drugs (including carbamazepine, valproate, lamotrigine, and quetiapine), had significantly higher concentrations of TSH and fT4 and lower concentrations of fT3 [55]. This reinforces the need for careful monitoring of thyroid parameters in this population and highlights the cost–benefit decision on whether to adjust hormone levels with levothyroxine or—rarely—stop lithium. The results of differences in thyroid function were not seen in a large population‐based study that compared lithium users to a matched cohort of valproate users [57]. While the primary focus was on the frequency of lab monitoring rather than clinical tolerability, the researchers utilized valproate as a comparator because it is a common alternative for BD. While lithium users had statistically higher rates of creatinine and TSH testing than valproate users, the absolute differences were not clinically meaningful.

### 4.7 Sex and Gender Differences in Lithium Levels

When analyzing sex or gender differences for lithium levels, in a study of 501 psychiatric patients [58], men exhibited lithium levels approximately 0.07 mmol/L lower than women. The researchers suggested this difference might be due to physiological factors, noting that men generally have greater lean mass and less fat mass than women. These results were not supported by a larger analysis of 7449 lithium blood samples over a five‐year period [48], where no statistically significant difference in lithium concentrations was found between males and females (0.51 mmol/L and 0.52 mmol/L, respectively).

## 5 Limitations

This review is subject to several limitations that should be considered when interpreting its findings. A narrative synthesis approach was employed, given the substantial heterogeneity in study designs, outcome definitions, and lithium measurement methods, which prevented statistical pooling. Importantly, all studies employed observational designs (retrospective cohort, longitudinal cohort, or cross‐sectional). This renders causal inference impossible regarding optimal therapeutic targets and constrains the strength of conclusions that can be drawn about dose–response relationships or the equilibrium between optimizing dose and tolerability. Extending this concern, the predominantly retrospective nature of the studies introduces bias inherent to retrospective data collection. In particular, the exploratory design of drug interaction studies means that observed associations between specific drug combinations and lithium level elevations cannot be interpreted as confirmatory evidence of causation. A further limitation is the lack of detailed clinical information, including psychiatric diagnoses, treatment indications, and the severity of comorbid conditions. Moreover, the diagnostic heterogeneity across some studies—encompassing both BD and other conditions such as MDD or schizoaffective disorder—while reflective of real‐world clinical practice, may contribute to variability in reported lithium levels. Additionally, several studies reported demographic and clinical characteristics (e.g., mean age, sex distribution) for the total study sample but did not carry out a stratified analysis for the older adult subgroup. In cases where only total‐sample data were available, data were included with appropriate caution, which may have introduced some imprecision in estimates pertaining specifically to older adults. Compounding this, these studies applied inconsistent age thresholds to define “older adults,” with cut‐offs ranging from ≥ 50 to ≥ 65 years. Several studies encompassed a broad age range without providing separate analyses for the older adult subgroup, compromising cross‐study comparability and reducing the specificity of conclusions drawn about this population. Of particular concern, the evidence base for the oldest‐old subgroup is notably sparse. No study provided a separate analysis for this age group, representing a critical evidence gap given the pharmacokinetic vulnerability and polypharmacy burden in this population [77].

A further methodological concern relates to the timing of serum lithium sampling relative to the last dose. The standard 12‐h post‐dose trough measurement is recommended for consistent interpretation, yet sampling timing was inconsistently reported across studies, which may have contributed meaningfully to variability in observed lithium concentrations independently of true clinical differences. Studies relied on routine clinical procedures rather than structured research protocols and they frequently lacked validated instruments to systematically evaluate treatment response or side effects, thereby constraining the comparability and depth of tolerability data across studies. Moreover, the marked sex distribution imbalance across included studies—ranging from 4% to 92% women—does not allow for conclusions regarding sex‐specific therapeutic targets or tolerability profiles in older adults and undermines the generalizability of findings across sexes. Selection bias represents an additional concern across several study types. In discontinuation studies, patients who ceased lithium treatment may have had inherently different clinical trajectories—such as steeper renal decline—compared to those who continued, potentially skewing findings on tolerability and safety. Furthermore, clinicians may systematically avoid prescribing lithium to patients with mild cognitive complaints as this may impact their medication adherence, which could introduce bias into studies examining neuroprotective effects and lead to an overestimation of lithium's protective associations.

As a formal meta‐analysis was not conducted, assessment of publication bias was not possible, and it is possible that studies with unremarkable or null findings were less likely to be published, potentially influencing the observed distribution of reported levels. Furthermore, studies were largely concentrated in Europe (*n* = 12), with only two conducted in North America (United States and Canada), and were conducted across heterogeneous clinical settings including community, ambulatory, and tertiary care contexts. Given that lithium levels, monitoring practices, and clinical outcomes may differ substantially across these settings, synthesizing findings without accounting for setting‐level variation may introduce additional imprecision. No studies from Asia, Africa, South America, or Oceania met the inclusion criteria, substantially limiting the applicability of findings to diverse global populations. Restricting the search to English‐language publications may have introduced language bias and excluded relevant evidence from non‐English‐speaking contexts, further compounding the geographic representational gap.

## 6 Conclusion

This systematic review supports age‐adjusted lithium therapeutic targets for OABD. The observed serum levels were substantially lower than standard laboratory reference ranges and consistent with existing expert consensus recommendations. Renal function, polypharmacy—particularly with NSAIDs, and medications for high blood pressure like diuretics and ACE inhibitors—and suboptimal monitoring compliance across care settings emerged as the most clinically significant and modifiable determinants of lithium safety in this population. These findings complement existing expert consensus recommendations for individualized monitoring and are consistent with calls for clinical laboratories to adopt age‐stratified lithium reference ranges. Whether such adoption translates to measurable improvements in patient safety outcomes remains an important question for implementation research.

The evidence base remains constrained by the exclusively observational nature of included studies, substantial heterogeneity in design and outcome reporting, geographic concentration in European settings, and a near‐complete absence of data for the oldest‐old subgroup—limitations that temper the strength of conclusions that can be drawn.

Future research should prioritize prospective longitudinal designs, inclusion of the oldest‐old and more diverse global populations, and implementation studies evaluating the real‐world uptake of age‐specific laboratory reference ranges. There is a need for studies reporting serum lithium levels in mmol/L alongside oral daily doses in mg across different treatment phases. Coordinated action across clinical, laboratory, and health systems levels will be important to translate the available evidence into practice, with the goal of promoting safer and more effective lithium management for OABD.

Implementation research is equally needed, including evaluation of clinician education strategies and automated monitoring reminders to improve adherence to monitoring guidelines. Most critically, coordinated initiatives are needed to promote the adoption of age‐stratified lithium therapeutic reference ranges in clinical laboratories globally, translating the evidence synthesized in this and prior reviews into actionable clinical practice change.

## Disclosure

Eduard Vieta has received grants and served as a consultant, advisor, or CME speaker for the following entities: AB‐Biotics, Abbott, AbbVie, Adamed, Alcediag, Angelini, Biogen, Beckley‐Psytech, Biohaven, Boehringer‐Ingelheim, Bristol‐Myers‐Squibb, Casen‐Recordati, Celon Pharma, Clariane, Compass, Dainippon Sumitomo Pharma, Eli Lilly, Esteve, Ethypharm, Ferrer, Gedeon Richter, GH Research, Glaxo‐Smith Kline, HMNC, Intra‐Cellular therapies, Idorsia, Johnson & Johnson, LB Pharma, Lundbeck, Luye Pharma, Medincell, Merck, Mitsubishi Tanabe Pharma, Newraxpharm, Newron, Novartis, Organon, Orion Corporation, Otsuka, Roche, Rovi, Sage, Sanofi‐Aventis, Sunovion, Takeda, Teva, and Viatris, outside the submitted work. Lars Vedel Kessing has within the recent 3 years served as a consultant for Lundbeck and Teva.

Hilary Blumberg has served as a consultant to Lilly, Biohaven, and Boehringer‐Ingelheim.

## Supporting information


**Data S1:** Supporting Information.

## Data Availability

Data sharing not applicable to this article as no datasets were generated or analysed during the current study.
