# Supplementary material for: Towards a Safe and Effective Lithium Therapeutic Range for Older Adults With Bipolar Disorder: An ISBD Task Force Systematic Review
Source: Bipolar Disord. 2026 Jul 16;28(5):e70151. doi: 10.1111/bdi.70151 (PMC13373820; doi:10.1111/bdi.70151)
Supplement: Supplementary file 1 — Data S1: Supporting Information. [file BDI-28-0-s001.docx]

### Supplemental Files:

Inclusion criteria:

Species: Human

Age: 60+ (either separate analysis or correction for age or average age >60

Population: Bipolar disorder

Full peer reviewed text published (exclude comments, editorials, single case report, conference abstracts)

**Database:**

Ovid MEDLINE(R) ALL <1946 to May 22, 2025>

| **#** | **Query** | **Results from 26 May 2025** |
| --- | --- | --- |
| 1 | Lithium Carbonate/ or Lithium/ | 25,916 |
| 2 | lithium.mp. | 82,266 |
| 3 | 1 or 2 | 82,266 |
| 4 | exp "Bipolar and Related Disorders"/ | 47,422 |
| 5 | bipolar disorder*.mp. | 62,285 |
| 6 | 4 or 5 | 62,293 |
| 7 | Middle Aged/ or Aged/ or "Aged, 80 and over"/ | 5,913,544 |
| 8 | (middle age* or elder* or senior* or older adult* or geriatric*).mp. | 5,492,901 |
| 9 | 7 or 8 | 6,122,257 |
| 10 | 3 and 6 and 9 | 3,259 |
| 11 | limit 10 to yr="2017 -Current" | 452 |
| 12 | limit 11 to English language | 432 |

Embase <1974 to 2025 May 28>

| **#** | **Query** | **Results from 29 May 2025** |
| --- | --- | --- |
| 1 | lithium/ or lithium carbonate/ | 75,164 |
| 2 | lithium.mp. | 120,448 |
| 3 | 1 or 2 | 120,448 |
| 4 | bipolar disorder/ | 87,019 |
| 5 | bipolar disorder*.mp. | 99,536 |
| 6 | 4 or 5 | 99,536 |
| 7 | aged/ | 4,173,928 |
| 8 | (elder* or senior* or older adult* or geriatric*).mp. | 1,104,726 |
| 9 | 7 or 8 | 4,449,517 |
| 10 | 3 and 6 and 9 | 2,056 |
| 11 | limit 10 to yr="2017 -Current" | 723 |
| 12 | limit 11 to english language | 715 |

**Database:**
EBM Reviews - Cochrane Central Register of Controlled Trials <April 2025>

| **#** | **Query** | **Results from 29 May 2025** |
| --- | --- | --- |
| 1 | Lithium Carbonate/ or Lithium/ | 1,196 |
| 2 | lithium.mp. | 3,400 |
| 3 | Bipolar Disorder/ | 3,587 |
| 4 | bipolar disorder*.mp. | 6,962 |
| 5 | 1 or 2 | 3,400 |
| 6 | 3 or 4 | 6,962 |
| 7 | "Aged, 80 and over"/ or Aged/ | 278,253 |
| 8 | (elder*or senior* or older adult* or geriatric*).mp. | 31,074 |
| 9 | 7 or 8 | 295,624 |
| 10 | 5 and 6 and 9 | 183 |
| 11 | limit 10 to yr="2017 -Current" | 20 |
| 12 | limit 11 to english language | 20 |
